# Tomato fruits expressing a bacterial feedback-insensitive 3-deoxy-d-arabino-heptulosonate 7-phosphate synthase of the shikimate pathway possess enhanced levels of multiple specialized metabolites and upgraded aroma

**DOI:** 10.1093/jxb/ert250

**Published:** 2013-09-04

**Authors:** Vered Tzin, Ilana Rogachev, Sagit Meir, Michal Moyal Ben Zvi, Tania Masci, Alexander Vainstein, Asaph Aharoni, Gad Galili

**Affiliations:** ^1^Department of Plant Sciences, Weizmann Institute of Science, PO Box 26, Rehovot 76100, Israel; ^2^Institute of Plant Sciences and Genetics in Agriculture, Faculty of Agriculture, The Hebrew University of Jerusalem, PO Box 12, Rehovot 76100, Israel

**Keywords:** Aromatic amino acids, 3-deoxy-d-arabino-heptulosonate 7-phosphate synthase, metabolism, shikimate pathway, tomato, volatiles.

## Abstract

Tomato (*Solanum lycopersicum*) fruit contains significant amounts of bioactive compounds, particularly multiple classes of specialized metabolites. Enhancing the synthesis and accumulation of these substances, specifically in fruits, are central for improving tomato fruit quality (e.g. flavour and aroma) and could aid in elucidate pathways of specialized metabolism. To promote the production of specialized metabolites in tomato fruit, this work expressed under a fruit ripening-specific promoter, E8, a bacterial *AroG* gene encoding a 3-deoxy-d-arabino-heptulosonate 7-phosphate synthase (DAHPS), which is feedback-insensitive to phenylalanine inhibition. DAHPS, the first enzyme of the shikimate pathway, links between the primary and specialized metabolism derived from aromatic amino acids. *AroG* expression influenced the levels of number of primary metabolites, such as shikimic acid and aromatic amino acids, as well as multiple volatile and non-volatile phenylpropanoids specialized metabolites and carotenoids. An organoleptic test, performed by trained panellists, suggested that the ripe *AroG*-expressing tomato fruits had a preferred floral aroma compare with fruits of the wild-type line. These results imply that fruit-specific manipulation of the conversion of primary to specialized metabolism is an attractive approach for improving fruit aroma and flavour qualities as well as discovering novel fruit-specialized metabolites.

## Introduction

Diets rich in fruits and vegetables are shown to be associated with the reduced incidence of chronic disease (Key *et al.*, 2002). These findings have led several governments to recommend and encourage the consumption of at least five portions of fruits and vegetables per day (Cooper, 2004). The health benefits conferred by certain fruits and vegetables have been generally attributed to the presence of health-promoting phytochemicals with potent antioxidant properties (also termed bioactives), such as carotenoids, phenylpropanoids, tocopherols and tocotrienols (vitamin E forms), and ascorbic acid (vitamin C). Ripe red tomato fruit contains significant amounts of these compounds and are the principal source of the carotenoid lycopene in the human diet (Enfissi *et al.*, 2010). In addition, they contain an estimated 30 volatile compounds that determine their flavour and aroma properties and are present in sufficient quantities to noticeably stimulate the olfactory system (Buttery and Ling, 1993; Ibdah *et al.*, 2006; Klee, 2010; Dal Cin *et al.*, 2011; Klee and Giovannoni, 2011). A significant part of the volatile as well as non-volatile specialized metabolites produced in tomato fruit are synthesized from the aromatic amino acids phenylalanine (Phe) and tyrosine (Tyr). The synthesis of these metabolites is up-regulated during fruit ripening and they therefore contribute to the unique flavour of tomato fruit (Baldwin *et al.*, 2000; White, 2002; Klee and Giovannoni, 2011).

To date, only limited amount of studies described the elucidation and manipulation of metabolic regulatory bottlenecks in the conversion of primary metabolism into aromatic specialized metabolites, with major efforts being directed at the production of Phe-derived volatiles (Dudareva and Pichersky, 2008). Examples of these studies include the aromatic l-amino acid decarboxylases that catalyse the conversion of Phe to phenethylamine and Tyr to tyramine (Tieman *et al.*, 2006; Gutensohn *et al.*, 2011), phenylacetaldehyde synthase that functions in the formation of phenylacetaldehyde, a constituent of floral scent (Kaminaga *et al.*, 2006), and isoeugenol synthase 1 that catalyses the formation of isoeugenol in petunia (*Petunia hybrida*) (Dexter *et al.*, 2007). The co-regulation between pathways of specialized metabolism precursors, as in the case of aromatic amino acids, is also far from being understood (Doerfler *et al.*, 2013). Recently, several studies have elucidated this metabolic link: (i) a gene of Phe biosynthesis, named *arogenate dehydratase1*, was recently characterized in petunia petals and its suppression resulted in varying degrees of reduction in the emission of different phenylpropanoids/benzenoid volatiles (Maeda *et al.*, 2010); and (ii) a number of transcription factors have been identified that regulate the production of volatile and no-volatile specialized metabolites in various plant species, such as petunia flowers and tomato fruits (Verdonk *et al.*, 2005; Dal Cin *et al.*, 2011; Moyal Ben Zvi *et al.*, 2012; Spitzer-Rimon *et al.*, 2012).

**Fig. 1.**
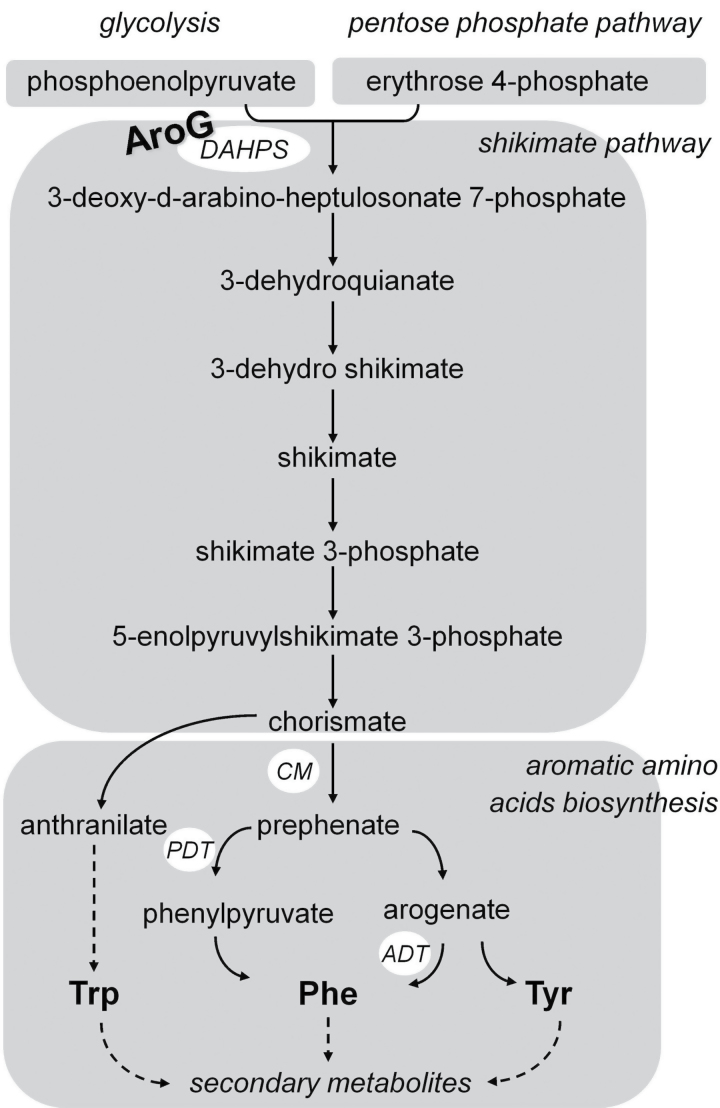
Schematic diagram of the shikimate pathway aromatic amino acid biosynthesis in plants. Only some enzymes are illustrated. Dashed arrows represent several enzymic steps. ADT, arogenate dehydratase; CM, chorismate mutase; DAHPS, 3-deoxy-d-arabino-2-heptulosonate 7-phosphate synthase; PDT, prephenate dehydretase.

To study the regulatory interaction between pathways of primary and specialized metabolism associated with the three aromatic amino acids [AAAs; Phe, Tyr, and tryptophan (Trp)], this work independently expressed in transgenic *Arabidopsis* (*Arabidopsis thaliana*) bacterial genes encoding: (i) a mutant feedback-insensitive chorismate mutase/prephenate dehydretase, the product of a bacterial *PheA*, associated with the conversion of chorismate to phenylpyruvate and Phe (Tzin *et al.*, 2009); and (ii) a mutant feedback-insensitive 3-deoxy-d-arabino-heptulosonate 7-phosphate synthase, the product of a bacterial *AroG*, encoding DAHPS, the first enzyme of the shikimate pathway (Tzin *et al.*, 2012; Fig. 1). Plants expressing these bacterial genes exhibited enhanced levels of a number of aromatic specialized metabolites in a manner that was specific to the bacterial enzyme (Tzin *et al.*, 2009, 2012).

To elucidate the impact of the shikimate pathway on the production of specialized metabolites in tomato fruits, this work expressed a bacterial Phe-feedback-insensitive AroG under a fruit-specific promoter in transgenic tomato plants. Fruits of these tomato plants exhibited altered levels of various primary metabolites as well as multiple volatile and non-volatile specialized metabolites, many of which are novel metabolites that have not yet been characterized and thus can serve as candidates for future metabolite discoveries. The results imply that fruit-specific manipulation of a gene that encodes an enzyme controlling the conversion of primary to specialized metabolism is an attractive tool for improving fruit aroma and flavour qualities. Such an approach may aid in overcoming the genetic linkage between long-shelf life and low flavour in tomato fruit, an issue that has so far been hard to solve by classical breeding.

## Materials and methods

### Plasmid construction

The truncated coding DNA sequence of *Escherichia coli AroG* was amplified by PCR with the following oligonucleotides: forward 5′-CAT*GCATGC*TGATGAATTATCAGAACGACGA-3′, which introduces a *Sph*I restriction site (underlined); and reverse 5′-G*GAATTC*CCCGCGACGCGCTTTTACTG-3′, which introduces an *Eco*RI restriction site (underlined). Two point-mutation forms of *AroG* encoding to feedback-insensitive proteins were constructed: *AroG175* with a point mutation at position 524 (Leu175Glu) and *AroG209* which has a point mutation at position 625-626 (Phe209Ala). The following PCR primers were used: AroG175 forward 5′-GTGCACCGCGAACAGGCATCAGGGCTT-3′ and reverse 5′-AAGCCCTGATGCCTGTTCGCGGTGCAC-3′ and AroG209 forward 5′-GCGCCGCACTGCGCCCTG TCCGTAACG-3′ and reverse 5′-CGTTACGGACAGGGCG CAGTGCGGCGC-3′. The RUBISCO small subunit-3A plastid transit peptide used (Shaul and Galili, 1993) was fused in frame to the 5′-end of the open reading frame. The genes were fused to the E8 promoter, which is spatially and temporally regulated in mature tomato fruit (*Solanum lycopersicum*)(Zhao *et al.*, 2009) and regulated by ethylene regulatory regions in a fruit-specific promoter (Deikman *et al.*, 1992). The genes were fused to three copies of a HA epitope tag fused to an octopine synthase terminator, and the entire fragment was subcloned into the Ti plasmid pART27 (Gleave, 1992). The chimeric genes were introduced into *Agrobacterium tumefaciens* EHA-105.

## Tomato stable transformation

Wild-type (WT; M82 cultivar) tomato plants were inoculated by submerging cotyledons in the transformed *A. tumefaciens* culture as previously described (Fillati *et al.*, 1987).

## Tomato growth and sampling

Tomato transformation and genotyping performed by Hazera Genetics (www.hazera.co.il). Flowers of greenhouse-grown plants were marked at anthesis, and fruits were harvested according to appearance and days post anthesis (DPA): ~42 DPA, mature green; ~44 DPA, breaker; and ~48 DPA, ripe red. Each biological repeat was a mixture of three to five individual fruits from the same stage of development. Immediately upon harvesting, peel and flesh (without the gel and seeds) were manually dissected and frozen in liquid nitrogen (Adato *et al.*, 2009).

## Immunoblot analysis

Immunoblots were performed as previously described (Stepansky and Galili, 2003) using monoclonal anti-haemagglutinin antibodies (Sigma Aldrich).

## Isoprenoid extraction and analysis

Isoprenoids were extracted from 100mg fresh weight of frozen tomato powder and analysed by HPLC with a photo diode array (Fraser *et al.*, 2000) with several modifications as previously published (Adato *et al.*, 2009).

## Metabolomics analysis by the use of UPLC/qTOF-MS

Non-targeted metabolic analysis was performed with aerial tissues of tomato 500mg peel or flesh extracted in 80% methanol. Sample preparation and injection conditions were performed as previously described (Mintz-Oron *et al.*, 2008). The analysis of the raw UPLC/qTOF-MS data was done using the XCMS software that performs chromatogram alignment, mass signal detection, and peak integration (Smith *et al.*, 2006) from the Bioconductor package version 2.1 for the R statistical language version 2.6.1. XCMS was used with the following parameters: fwhm = 10.8, step = 0.05, steps = 4, mzdiff = 0.07, snthresh = 8, max = 1000. Injections of samples in the positive and negative ionization modes were performed in separate injection sets and preprocessing was done for each ionization mode independently. Differential mass ions were determined using a Student’s t-test (JMP software).

## GC-MS extraction, derivatizion, and profiling of polar non-volatile extracts

GC-MS analysis of polar metabolites in the *AroG*_*209-9*_-overexpressing and WT fruit tissues (*n* = 5–6) were carried out as previously described (Mintz-Oron *et al.*, 2008). Xcalibur version 1.4 (Thermo Finnigan) was used for data analysis. Compounds were identified by comparison of their retention index and mass spectrum to those generated from authentic standards analysed on the same instrument. In cases when standards were not available, compounds were putatively identified by comparison of their retention index and mass spectrum to those present in the mass spectra library of Max-Planck-Institute for Plant Physiology, Golm, Germany (Q_MSRI_ID) and the commercial mass spectra library NIST05 (www.nist.gov). The response values for metabolites resulting from the Xcalibur processing method were normalized to the ribitol (adonitol, Sigma Aldrich) internal standard. For principal components analysis (PCA), the data were pretreated as follows: missing values for metabolites in one of the replicates were exchanged for the average between the replicates, zero values were replaced by a value 4-times lower than the minimal non-zero value in the data set, data were normalized to the mean of each metabolite across all samples and log-transformed (Tzin *et al.*, 2009). For statistical analysis of the whole data matrix, a two-way ANOVA test was performed with the two discriminating factors being line (WT or *AroG*_209-9_) and fruit developmental stage. Both PCA plots and the two-way ANOVA test were constructed with the TMEV software package (Saeed *et al.*, 2003).

## GC-MS extraction and profiling of volatile aroma compounds in tomato fruits

GC-MS analysis of polar volatile compounds in the *AroG*_*209-9*_- expressing and WT fruit tissues samples was carried out as previously described (Spitzer-Rimon *et al.*, 2010) with several modifications: each biological replicate, consisting of 10g tissue from a mix of 2–5 cut fruits, harvested at the ripening stage (flesh and peel) (*n* = 4). Tissue was extracted with 30ml MTBE/hexane (1:1) containing 2μg isobutylbenzene as an internal standard. Following overnight incubation with shaking at 150rpm, the extract was centrifuged at 10,500 *g* for 10min and the supernatant was passed through a 0.2 μm filter. Samples were evaporated, using liquid nitrogen, to a final volume of 200 μl before injection into a GC-MS instrument (Ben Zvi *et al.*, 2012). Further identification of the compounds was based on a comparison of mass spectra and retention times with those of authentic standards (Sigma Aldrich) analysed under similar conditions (Davidovich-Rikanati *et al.*, 2007).

## Sensory panel and evaluation of samples

A panel of 10 trained flavour specialists evaluated the aroma of samples by smelling the fruits. Preliminary tests were carried out to improve the ability of the assessors to recognize odour defects and consistently quantify sensory properties. The panellists had previously been trained in the quantitative description of tomato attributes according to selection trials based on French norms (ISO8586-1, AFNOR V09-003). For each fruit sample, cut sections containing all fruit tissues were used for aroma evaluation by the panel. Several attributes were chosen: acidic, floral, fresh, green, metallic, musty, ripe, spicy, and sweet in addition to global aroma intensity. Values of individual fruits (*n* = 3–4) were ranked between 0 (none) to 5 (very strong). When completed, the panel members discussed their scores and agreed on the final summarizing score of each aroma group.

## Results

### Generation of transgenic tomato plants expressing a bacterial feedback-insensitive 3-deoxy-d-arabino-heptulosonate 7-phosphate

**Fig. 2.**
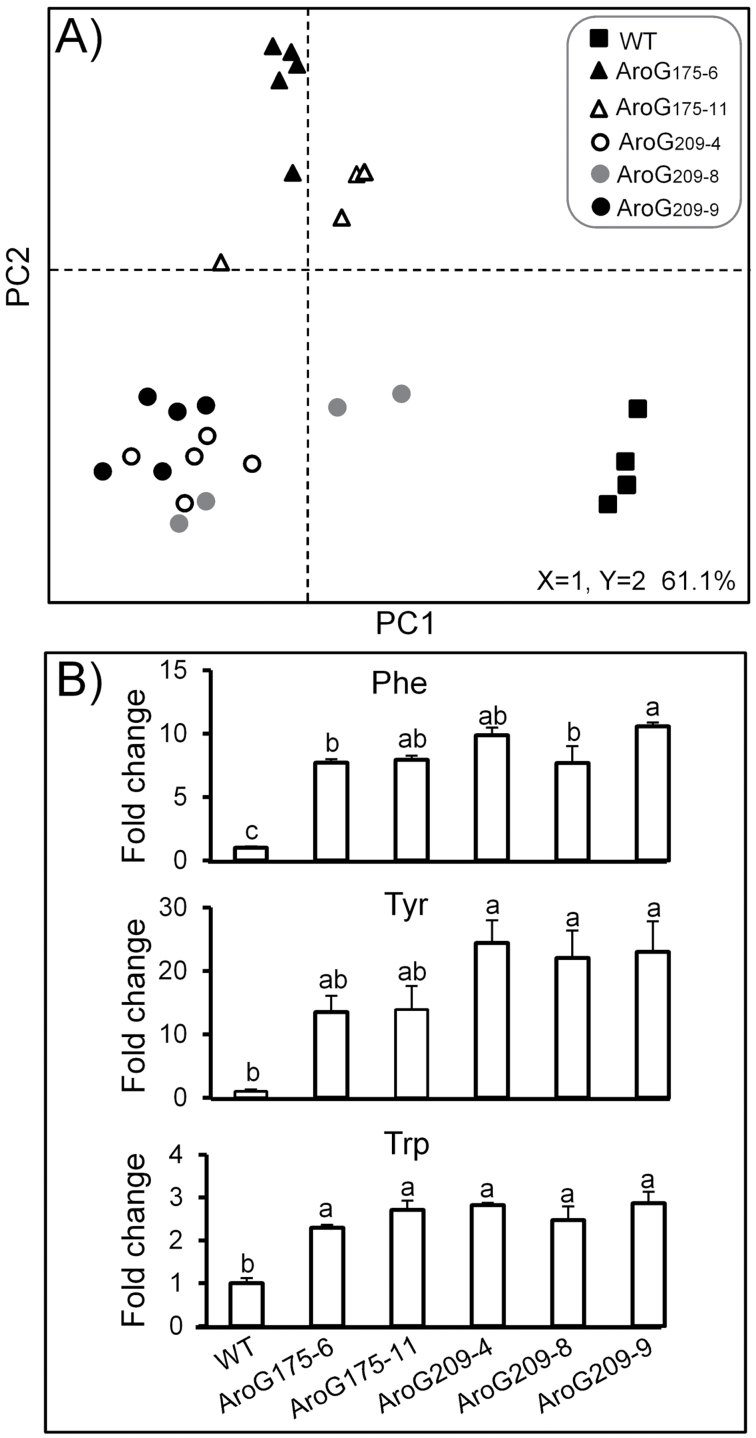
Metabolic profiling of transgenic tomato plants expressing a bacterial feedback-insensitive *AroG* gene. Samples of T_1_-generation ripe red tomato fruits were collected, extracted, and analysed using the high-resolution LC-MS metabolomics platform. The independent transgenic lines are AroG_175-6_, AroG_175-11_, AroG_209-4_, AroG_209-8_, and AroG_209-9_. (A) PCA plot of datasets was obtained from 3094 mass signals in the negative ionization mode; each data point represents an independent sample (*n* = 5 for each line); the first two components are given in this panel. (B) Relative levels of the three aromatic amino acids; asterisks indicate statistically significant differences between the *AroG* lines and the wild-type (WT), using ANOVA; bars indicate standard errors.

To evaluate the impact of enhancing metabolic flow through the shikimate pathway on the accumulation of primary and specialized metabolites in tomato fruits, this work expressed two forms of a bacterial gene encoding two variants of a feedback-insensitive bacterial AroG enzyme, *AroG*_*175*_ or *AroG*_*209*_ (Tzin *et al.*, 2012). These two variants were expressed under the control of the fruit ripening-specific E8 promoter which is induced by ethylene upon fruit maturation (Deikman *et al.*, 1992; Zhao *et al.*, 2009). The transgenic *AroG*_*175*_- and *AroG*_*209*_ *-expressing* plants had comparable phenotypes to the WT plants and were fully fertile (data not shown). A schematic diagram of the *AroG* constructs introduced to tomato plants and expression of the AroG proteins in ripe red fruit are presented in Supplementary Fig. S1 (available at *JXB* online). Ripe fruit were harvested from five independently transformed *AroG*_*175*_ (two lines) and *AroG*_*209*_ lines (three lines) and WT plants, and were analysed by an established high-resolution LC-MS-based metabolomics platform (negative ion-mode; Rogachev and Aharoni, 2012). PCA of whole ripe fruit extracts showed that the metabolic profile of the WT differed from those of the *AroG*_*175*_ and *AroG*_*209*_ lines (Fig. 2A). In addition, a clear difference was evident between the group of independently transformed *AroG*_*175*_ lines (*AroG*_*175-6*_ and *AroG*_*175-11*_) and *AroG*_*209*_ lines (*AroG*_*209-4*_, *AroG*_*209-8*_, and *AroG*_*209-9*_). This separation implied an influence of both the different mutations within the *AroG* genes as well as the level of expression of the same transgene in different transformed plants on the extent of the metabolic changes. The levels of all three AAAs were significantly increased in all five lines (Fig. 2B). Tyrosine level was also significantly higher in the *AroG*_*175*_ lines and the highest in the *AroG*_*209*_ lines as compared to the WT. For further study, the homozygous *AroG*_*209-9*_ line, which contained a single insertion, was selected.

### Effect of AroG_209-9_ expression on the levels of non-volatile metabolites accumulating during fruit maturation

The fleshy fruit of tomato develops in several marked stages, each possessing a characteristic metabolic profile (Carrari *et al.*, 2006). In view of that, fruits were separated into two tissues: (i) peel, which is typically composed of multiple cell types, including epidermis, collenchyma, and some parenchyma, and (ii) flesh, which refers to the pericarp material from which the peel has been removed and therefore is predominantly composed of parenchyma and collenchyma (Mintz-Oron *et al.*, 2008). Tomato fruit development can be divided into four main phases: cell differentiation, cell division, cell expansion, and ripening (Gillaspy *et al.*, 1993). The E8 promoter used to direct the expression of the *AroG*_*209-9*_ construct has been previously shown to direct a relatively minor expression starting at the mature green stage and become efficiently activated at the ripening stage (Lincoln and Fischer, 1988; Good *et al.*, 1994; Deikman *et al.*, 1998). Therefore, the current work examined the metabolic profiling of fruits at three ripening stages, namely mature green, breaker, and ripe red stages. In order to profile polar compounds, in particular primary metabolites, the previously established GC-MS analysis of derivatized fruit extracts was used (Fernie *et al.*, 2004). In tomato fruit, this technology allowed the monitoring of the levels of 64 metabolites, including amino acids, organic acids, sugar alcohols, tricarboxylic acid cycle intermediates, soluble sugars, sugar phosphates, and a few specialized metabolites (Supplementary Table S1). In addition, the developing tomato fruits were also subjected to high-resolution LC-MS analysis in both electrospray ionization-positive and -negative modes that covers mostly semi-polar, specialized metabolites (Supplementary Tables S2 and S3). This technology allowed the monitoring of the levels of 63 metabolites, including specialized metabolites derived from the phenylpropanoids, and steroidal alkaloids pathways as well as some primary metabolites. Finally, a stand-alone HPLC-PDA system coupled to UV and fluorescent detectors was employed for targeted analysis of nine isoprenoids, including carotenoids, chlorophylls, and tocopherols (Supplementary Table S4).

**Fig. 3.**
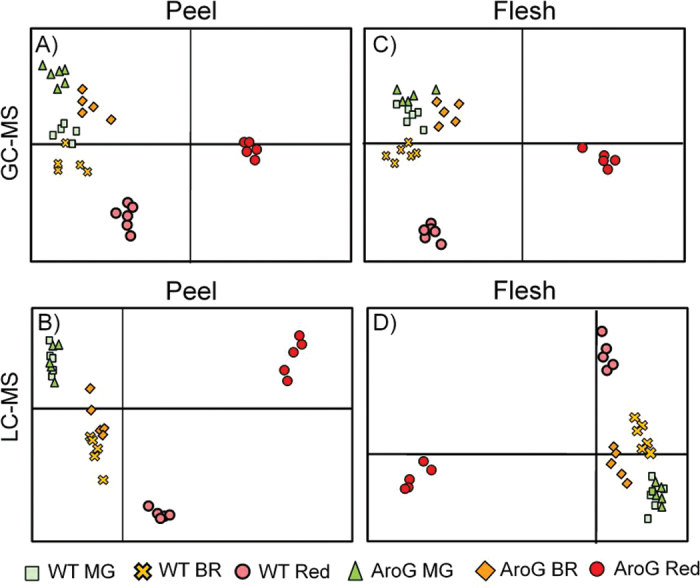
Metabolite profiles of developing tomato fruits of the *AroG*_*209-9*_ line and the wild type (WT). (A and C) PCA plots of metabolic profiles obtained by GC-MS analysis (125 detected metabolites) in peel (A) and flesh (C). (B and D) PCA plots of metabolic profiles obtained by LC-MS analysis in negative ion mode from 3931 mass signals in peel (B) and 1979 mass signals in flesh (D). T_2_-generation fruit were sampled in three developmental stages: mature green (MG, ~42 days post anthesis, DPA), breaker (BR, ~44 DPA), and Red (ripe red; ~48 DPA), and separated to peel and flesh (*n* = 5–6 for each line and developing stage) (this figure is available in colour at *JXB* online).

To obtain a broad view of the differences in the metabolites of fruit peel and flesh tissues, this study conducted PCA on the data sets derived from the metabolite profiling using GC-MS (Fig. 3A, C) and LC-MS (Fig. 3B, D). In these analyses, the *AroG*_*209-9*_ line and the WT were essentially grouped together at the mature green and breaker stages, while they were clearly separated at the ripe red stage in both the peel and flesh tissues. More detailed analysis of the LC-MS results revealed many more putative metabolites (approximately 220 in peel and 120 in flesh, with a mean of five mass signals per metabolite) whose levels were significantly different between fruits of the WT and the *AroG*_*209-9*_ line during the ripe red stage (for more details, see Venn diagram in Supplementary Fig. S2).

Subsequently, this work performed correlation coefficient analyses on 45 selected unknown mass signals whose levels were different between *AroG*_*209-9*_ and WT lines in the GC-MS and LC-MS analyses (Table 1). As shown in Supplementary Fig. S3, approximately 60 known metabolites and mass signals, including AAAs, shikimic acid, prephenic acid, oligosaccharides, and phenylpropanoids as well as an additional 43 unknown mass signals were highly clustered. These results implied that *AroG*_*209-9*_ expression has a major influence on the accumulation of primary and specialized metabolites in both the peel and flesh of the ripe red tomato fruit. This work also performed correlation coefficient analyses of GC-MS and LC-MS-identified metabolites and mass signals, which are presented as heat maps in Supplementary Fig. S4. Three major groups of compounds were highly clustered: group I that includes 12 compounds, mostly amino acids and monosaccharides; group II that includes 26 compounds, mostly organic acids, steroidal alkaloids and phenylpropanoids, and group III that includes 43 metabolites mostly AAAs, oligosaccharides and phenylpropanoids. This suggested strong cross-talk between the primary and the specialized metabolites.

**Table 1.** *Metabolites accumulating in the peel of developing tomato fruits expressing* AroG_209-9_

Metabolites	Peel		
	Mature green	Breaker	Ripe red
Amino acids			
Phe (G)	1.01±0.22	4.74±0.69^*a*^	88.95±8.88^*a*^
Tyr (G)	1.61±0.42	2.38±0.30	171.95±42.80^*a*^
Trp (G)	0.64±0.15	2.16±0.27	4.12±0.26^*a*^
Asn (G)	0.41±0.05^*a*^	0.52±0.11	0.51±0.07
Gln (G)	0.02±0.01^*a*^	0.02±0.01	0.39±0.05
Gly (G)	0.50±0.06^*a*^	1.30±0.23	1.69±0.25
N-acetyl-Glu (G)	0.03±0.00^*a*^	0.20±0.10	0.39±0.05
Thr (G)	0.47±0.05^*a*^	1.02±0.22	1.08±0.09
Organic acids			
Fumaric acid (G)	1.45±0.13	1.81±0.29	2.48±0.32^*a*^
Prephenic acid (G)	7.6±1.2^*a*^	354.3±119.9^*a*^	604.8±97.9^*a*^
Shikimic acid (G)	2.28±0.44	2.52±0.26^*a*^	17.43±0.83^*a*^
Sugars			
Cellobiose (G)	0.93±0.09	1.28±0.10	7.99±0.81^*a*^
Fructose (G)	1.74±0.10^*a*^	0.99±0.07	1.05±0.13
Fructose-6-P (G)	0.48±0.02^*a*^	0.83±0.07	0.91±0.13
Mannose (G)	1.69±0.13^*a*^	1.19±0.17	1.17±0.15
Raffinose (G)	1.24±0.25	2.00±0.21	103.1±17.2^*a*^
Trehalose (G)	0.54±0.09	0.67±0.18	73.6±6.1^*a*^
Polyamines			
Putrescine (G)	1.06±0.07	0.58±0.02	0.34±0.03^*a*^
Nucleosides			
Guanosine (G)	0.82±0.28	0.93±0.20	1.90±0.14^*a*^
Phenylpropanoids			
3-Caffeoylquinic acid (G)	0.89±0.18	1.30±0.21	2.86±0.24^*a*^
4-Caffeoylquinic acid (G)	0.67±0.11	0.98±0.25	2.14±0.09^*a*^
Coumaric acid (G)	0.70±0.10	2.66±0.62	164.8±18.4^*a*^
Coumaric acid-hexose I (L)	0.80±0.19	0.56±0.12	15.28±3.98^*a*^
Naringenin chalcone-hexose IV (L)	0.83±0.09	0.72±0.10	5.22±0.70^*a*^
Quercetin (L)	0.39±0.06^*a*^	0.69±0.13	2.60±0.89
Quercetin-hexose-deoxyhexose-pentose-*p*-coumaric acid (L)	0.99±0.11	1.09±0.32	3.85±0.30^*a*^
Tricaffeoylquinic acid (L)	0.94±0.16	0.69±0.18	2.33±0.18^*a*^
Carotenoids			
Phytoene (H)	ND	ND	0.21±0.09^*a*^
Phytofluene (H)	ND	ND	0.08±0.05^*a*^
Lycopene-like (H)	ND	ND	0.29±0.19^*a*^

Tomato fruits from T_3_ generation were used. Metabolite levels are presented as fold-change (*AroG*_*209-9*_/WT) in each developing stage: mean ± SE. ^*a*^ *P* < 0.05 (false discovery rate) as analysed by two-way ANOVA and a Student *t*-test. G, GC-MS (*n* = 6); H, HPLC-PDA (*n* = 4); L, UPLC/qTOF-MS (*n* = 5); ND, not detected.

### Effect of AroG_209-9_ expression on the levels of non-volatile metabolites in peel tissue during fruit development

Table 1 presents a list of annotated metabolites that were detected by the GC-MS, LC-MS, and HPLC-PDA analyses and possessed significantly altered levels in the *AroG*_*209-9*_ line compared to the WT in one or more stages of fruit development in peel. The major metabolic changes that were detected in the peel tissue at the ripe red stage included increased levels of primary metabolites associated with the shikimate pathway and AAA biosynthesis, including prephenic acid, shikimic acid, and the three AAAs. In addition, the levels of the three oligosaccharides cellobiose, raffinose, and trehalose as well as the nucleoside guanosine were increased in the *AroG*_*209-9*_ compared to the WT. The levels of several phenylpropanoids were higher in ripe red fruits of the *AroG*_*209-9*_ line including 3-caffeoylquinic acid, 4-caffeoylquinic acid, tricaffeoylquinic acid, coumaric acid, coumaric acid hexose I, naringenin chalcone hexose IV, and quercetin-hexose-deoxyhexose-pentose-*p*-coumaric acid. However, in the ripe red stage, the levels of only four metabolites were reduced including the polyamine putrescine and the carotenoids phytoene, phytofluene, and a lycopene-like metabolite. To summarize, levels of various known metabolites, particularly AAAs, organic acids, and phenylpropanoids, were increased in peel tissue during *AroG*_*209-9*_ fruit development.

### Effect of AroG_209-9_ expression on the levels of non-volatile metabolites in flesh tissue during fruit development

Table 2 presents the metabolites whose levels were significantly altered in the *AroG*_*209-9*_ line compared with the WT in the flesh tissue. The major metabolic changes detected in the flesh tissue of the ripe red stage *AroG*_*209-9*_ fruits included significantly increased levels of the primary metabolites prephenic acid, shikimic acid, Phe, and Tyr. In addition, the levels of three oligosaccharides—cellobiose, raffinose, and trehalose—were increased. In contrast, the levels of putrescine and three carotenoid compounds—phytoene, phytofluene and lycopene-like—were significantly decreased in the *AroG*_*209-9*_ fruits compared to the WT. Several flesh specific phenylpropanoids were increased in the ripe red stage, which includes coumaric acid, coumaric acid hexoside or derivative and kaempferol-glucose-rhamnose. Naringenin chalcone, a common flavanone in tomato, was significantly increased only in the breaker stage. In summary, the levels of several primary metabolites, Phe, Tyr, two organic acids associated with the shikimate pathway as well as multiple phenylpropanoids were higher in the flesh tissue along *AroG*_*209-9*_ fruit development compared to WT (total 19 compounds). There was also a considerable similarity in the metabolites whose levels were altered in both flesh and peel of the *AroG*_*209-9*_ fruit (total 14 compounds; Tables 1 and 2).

**Table 2.** *Metabolites accumulating in the flesh of developing tomato fruits expressing* AroG_209-9_

Metabolites	Flesh		
	Mature green	Breaker	Ripe red
Amino acids			
Phe (G)	0.88±0.10	2.41±0.26^*a*^	22.08±3.22^*a*^
Tyr (G)	0.87±0.12	2.69±0.38	15.27±3.19^*a*^
Asn (G)	0.60±0.06	0.94±0.12	0.49±0.06^*a*^
Ile (G)	1.03±0.14	1.08±0.12	1.97±0.11^*a*^
Organic acids			
Prephenic acid (G)	3.4±0.4^*a*^	276.3±90.9^*a*^	235.9±31.3^*a*^
Shikimic acid (G)	1.13±0.13	2.53±0.55	62.93±11.69^*a*^
Sugars			
Cellobiose (G)	0.96±0.07	1.15±0.05	22.0±3.1^*a*^
Maltose (G)	1.23±0.11	0.95±0.05	1.54±0.08^*a*^
Raffinose (G)	0.38±0.09	2.79±0.56	92.3±15.4^*a*^
Trehalose (G)	1.76±0.33	0.91±0.16	5.31±0.88^*a*^
Polyamines			
Putrescine (G)	0.83±0.06	0.79±0.11	0.16±0.01^*a*^
Phenylpropanoids			
Coumaric acid (G)	0.35±0.05	2.62±0.68	613.6±54.2^*a*^
Coumaric acid hexoside or derivative (L)	1.07±0.18	1.84±0.29	114.5±15.27^*a*^
Kaempferol-glucose-rhamnose (L)	1.09±0.10	0.92±0.04	108.6±27.70^*a*^
Naringenin chalcone (L)	0.51±0.14	10.08±2.91^*a*^	1.78±0.42
Quercetin (L)	0.23±0.03^*a*^	0.72±0.14	1.13±0.19
Carotenoids			
Phytoene (H)	ND	ND	>0.001^*a*^
Phytofluene (H)	ND	ND	>0.001^*a*^
Lycopene-like (H)	ND	ND	0.55±0.10^*a*^

Tomato fruits from T_3_ generation were used. Metabolite levels are presented as fold-change (*AroG*_*209-9*_/WT) in each developing stage: mean ± SE. ^*a*^ *P* < 0.05 (false discovery rate) as analysed by two-way ANOVA and a Student *t*-test. G, GC-MS (*n* = 6); H, HPLC-PDA (*n* = 4); L, UPLC/qTOF-MS (*n* = 5); ND, not detected.

### Effect of AroG_209_ expression on aroma-associated volatile metabolites and aroma in ripe red fruit

**Fig. 4.**
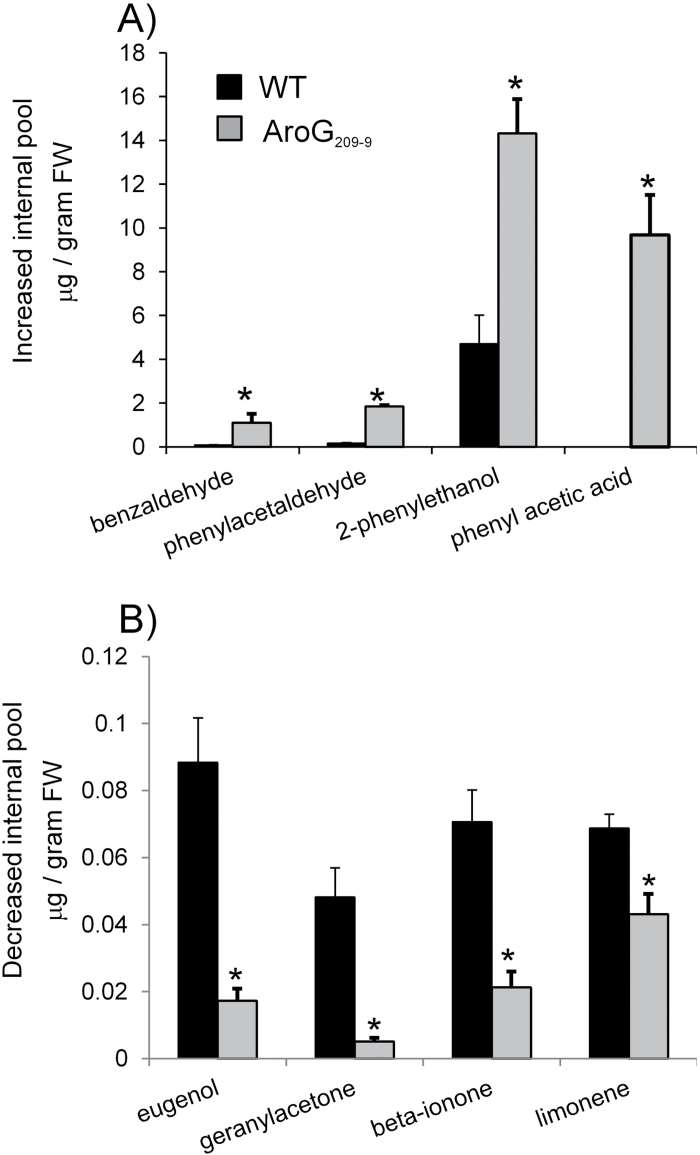
Internal pools of volatile compounds altered in ripe red tomato fruits expressing *AroG*_*209-9*_. Volatile levels increased (A) and decreased (B) in the *AroG*_*209-9*_-expressing line. Asterisks indicate statistically significant differences between the *AroG*_*209-9*_ line and the wild type (WT), using Student’s t-test. Bars indicate standard errors (*n* = 4). Tomato fruits from T_3_ generation were used.

**Fig. 5.**
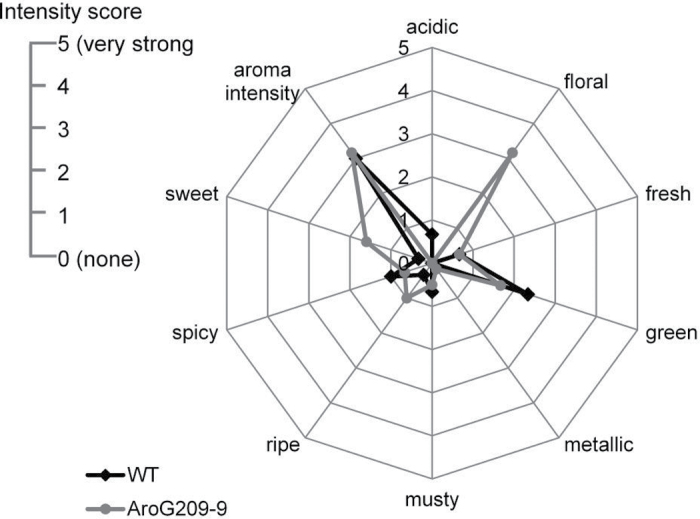
A sensory profile of red ripe tomatoes expressing the *AroG*_*209-9*_ gene. Each descriptor was scored on a 0 to 5 point scale. Tomato fruits from T_3_ generation were used.

In order to analyse the levels of volatiles in ripe red fruits of *AroG*_*209-9*-_expressing tomato plants, volatile metabolites were detected using GC-MS. The levels of the Phe-derived volatiles (benzaldehyde, phenylacetaldehyde, 2-phenylethanol, and phenylacetic acid) were higher in the ripe red fruits of the *AroG*_*209-9*_ line compared to the WT (Fig. 4A), while the levels of eugenol (a catabolic product of *p*-coumaric acid) and three terpenoids (geranylacetone, β-ionone, and limonene) were lower in the ripe red fruits of the *AroG*_*209-9*_ line compared to the WT (Fig. 4B). Based on these results, ripe red fruits of the *AroG*_*209-9*_ line were subjected further to an organoleptic test to test the impact of the metabolic changes on fruit aroma characteristics. The choice of an organoleptic test was because chemical analysis of flavour compounds provides relatively little insight into the actual flavour experience, while sensory attributes, preferences, and decisions can be statistically related to chemical components in foods (Martens *et al.*, 1994). Correlation of physical measurements with sensory analysis also provides some meaning to instrumental data, as was shown with apple and tomato (Baldwin *et al.*, 1998). The organoleptic test was performed by a dedicated panel of professionals trained in the quantitative description of tomato attributes according to selection trials, based on their own designated protocol. Whole ripe red fruits from each line were cut into two slices and evaluated by the various panel members through sniffing the samples (*n* = 3–4 biological repeats). Several professional attributes were considered, namely acidic, floral, fresh, green, metallic, musty, ripe, spicy, and sweet in addition to global aroma intensity. Taken together, the organoleptic panel suggested that the fruit of the *AroG*_*209-9*_ line possessed more ‘floral’ aroma properties than the fruit of the WT tomato line (Fig. 5).

## Discussion

### Expression of a bacterial feedback-insensitive AroG enzyme leads to large metabolic changes in tomato fruit

**Fig. 6.**
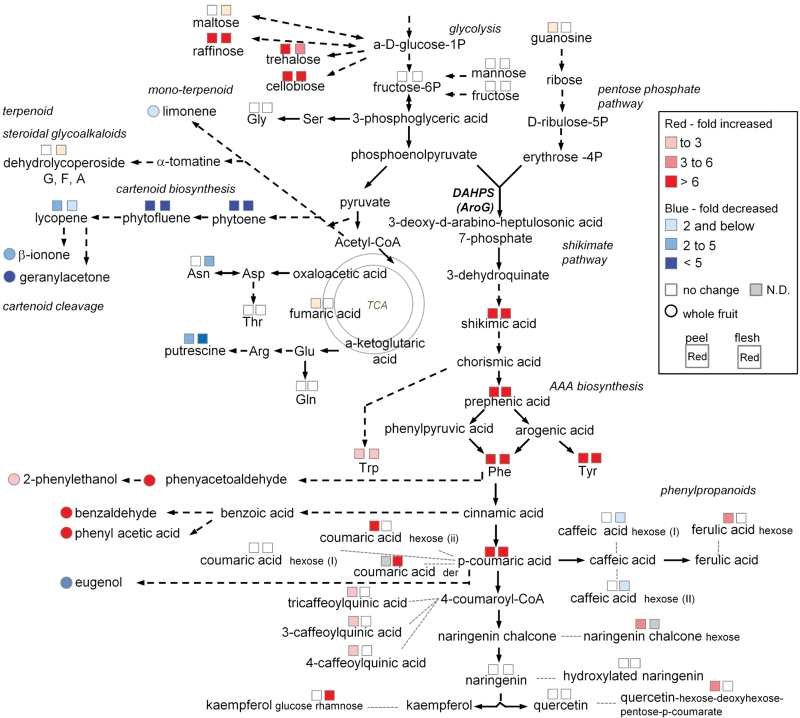
A metabolic pathway scheme summarizing the metabolic changes in ripe red tomato fruits expressing the *AroG*_*209-9*_ gene. Metabolites whose levels significantly increased or decreased in the transgenic fruits (peel and flesh), compared to the WT, are marked in red (fold-increased) or blue (fold-decreased). Circles mark the volatiles that were detected only in the ripe red developing stage. Broken arrows represent several consecutive enzymic steps.

Ripe red tomato fruits contain hundreds of volatile and non-volatile specialized metabolites, particularly phenylpropanoids and carotenoids, which determine their flavour and nutritional qualities (Enfissi *et al.*, 2010). Phenylpropanoids represent a major class of specialized metabolites accumulating in tomato fruit, which are produced via the shikimate and AAA biosynthesis pathways. The carotenoids and various volatile compounds derived from them are synthesized by a different metabolic pathway that may compete with the shikimate pathway on the glycolysis-associated metabolite phosphoenolpyruvate (PEP) (Negre-Zakharov *et al.*, 2009). The current work found that *AroG* gene expression, specifically in the ripe red tomato fruits, triggers significant increases in the levels of two primary metabolites of the shikimate pathway (shikimic acid and prephenic acid) and all three AAAs (Fig. 6). An analogous effect of the bacterial *AroG* on the levels of shikimate pathway metabolites had also been observed previously in *Arabidopsis* plants (Tzin *et al.*, 2012). Taken together, these results thus indicate regulation of the entire shikimate pathway. An example illustrating the complexity of the shikimate pathway was previously reported by Ding *et al.* (2007) who functionally analysed the impact of suppressed expression of the tobacco shikimate-pathway enzyme 3-dehydroquinate dehydratase/shikimate dehydrogenase in transgenic plants. They found that suppressing the level of this enzyme caused an unexpected increase, rather than decrease, in the level of shikimate, a metabolite downstream to that produced by the 3-dehydroquinate dehydratase/shikimate dehydrogenase enzyme.

In addition to shikimate, tomato fruits expressing the *AroG*_*209-9*_ gene possessed altered levels of other primary metabolites, namely sugars such as oligosaccharides (i.e. cellobiose, raffinose, and trehalose) and monosaccharides (i.e. fructose, fructose-6-phosphate, maltose, and mannose), nucleoside (guanosine) which degraded to d-ribose, which is one of the pentose phosphate pathway intermediates. Evidence from radiolabelling work in tomato fruit suggested that gluconeogenesis does occur during ripening stages, when sugars are accumulating rapidly (Farineau and Lavalmartin, 1977; Halinska and Frenkel, 1991; Osorio *et al.*, 2013). This may cause an increase in the oligosucchride levels in the AroG_209-9_ line. The levels of several amino acids, namely Phe, Tyr, Trp, Asn, Gln, Gly, Ile, N-acetyl-Glu, and Thr as well as the polyamine putrescine and the tricarboxylic acid cycle metabolite fumaric acid were also altered in the *AroG*_*209-9*_ line, compared to the control. These observations suggest the existence of a metabolic cross-interaction of the shikimate pathway with other pathways of primary metabolism in tomato fruits. The existence of a cross-regulation between different metabolic pathways in ripe red tomato fruits has been previously reported to occur between carotenoids and sugars (i.e. sucrose, glucose, and fructose) (Fraser *et al.*, 2007); polyamines and amino acids (Mattoo *et al.*, 2010); tricarboxylic acid cycle metabolites malate, starch, and sugars (Centeno *et al.*, 2011; Osorio *et al.*, 2013); as well as between fruit-surface cuticular lipids and the phenylpropanoids (Adato *et al.*, 2009). The major metabolic changes occur in the ripe red stage and this is apparently due to use of the E8 promoter, which is most active during the ripening stage (Good *et al.*, 1994), with slight expression initiating at the mature green stage and increasing expression during ripening (Lincoln and Fischer, 1988; Deikman *et al.*, 1998). This work thus suggests that the reduction in the levels of substrates of carotenoid biosynthesis resulting from *AroG* expression may be due to a metabolic shift channelling acetyl-CoA towards gluconeogenesis (for oligosaccharides production) and PEP (for the shikimate pathway and phenylpropanoids).

### Phenylpropanoids are a dominant class of specialized metabolites that are differentially produced in the peel and flesh of tomato fruits

Tomato is a model system for plants bearing fleshy fruits, whose metabolic status varies between different stages of fruit development and between different tissues (Carrari *et al.*, 2006; Osorio *et al.*, 2013). In mature red fruits, there were major metabolic changes in the peel and flesh of the *AroG*_*209-9*_ line compared with the WT. The main changes occurring in these two tissues were in primary metabolites, particularly AAAs, organic acids, oligosaccharides, and putrescine. However, major changes in the levels of diverse specialized metabolites, mostly phenylpropanoids, occurred in the peel. Naringenin chalcone-hexose, for instance, was detected only in the peel and highly accumulated during the ripe red stage. Previous reports showed that the peel of tomato fruits is enriched in a variety of phenylpropanoids metabolites compared to the flesh (Mintz-Oron *et al.*, 2008; Mounet *et al.*, 2009; Ballester *et al.*, 2010). While the secondary metabolites present in the peel can protect against changing environmental conditions and aid in deterring pathogens, they could also play a role in attracting seed-dispersing herbivores. Naringenin chalcone, accumulating in the fruit peel, could serve in the protection against UV radiation and in the attraction of herbivores by its intense pigmentation (Bovy *et al.*, 2002; Whitney, 2005) and could also serve as a structural element of the cuticle (Mintz-Oron *et al.*, 2008).

### AroG_209-9_ expression expands tomato fruit aroma

Tomato fruits contain a complex mixture of volatile and non-volatile compounds that contribute to the overall aroma and taste of the fruit. The volatile aroma compounds are essential for good tomato flavour (Baldwin *et al.*, 2000). These secondary metabolites are derived from a range of precursors, including carotenoids, lipids, and amino acids (Klee, 2010). Biosynthesis of volatile organic compounds depends on the availability of carbon, nitrogen, and sulphur as well as energy provided by primary metabolism (Dudareva *et al.*, 2013). The current results exposed several metabolic links between volatile and non-volatile compounds. For instance: reduction of the β-ionone and geranylacetone might be due to the reduction in the carotenoid levels detected (Simkin *et al.*, 2004), and the increase in Phe-derived volatiles (benzaldehyde, phenylacetaldehyde, 2-phenylethanol, and phenylacetic acid) might be due to an increase in Phe (Figs. 4 and 6). The metabolite analysis were supported by the human olfactory analysis. Since tomato fruit flavour involves an integration of sugars, acids, and a set of 30 or more key volatile chemicals (Klee, 2010), it is tempting to speculate that the increased levels of volatiles in the fruits of the *AroG*_*209-9*_ line is responsible for the altered aroma characteristic. 2-Phenylethanol, which was higher in the *AroG*_*209-9*_ line, is known to provide a sweet and fruity perception (Togari *et al.*, 1995; Zanor *et al.*, 2009) and may contribute to the ‘floral’ aroma. The reduction in the levels of the polyamine putrescine, which known to provides unpleasant aroma, may contribute for the better floral aroma.

In conclusion, this study provides new information on the regulation of the channelling of primary metabolism to specialized metabolism as well as the cross-interaction of the shikimate pathway with other metabolic pathways in tomato fruit. This research opens a new avenue to improve tomato fruit flavour without negatively influencing fruit shelf life, an issue that has so far been difficult to solve by classical breeding.

## Supplementary material

Supplementary data are available at *JXB* online.

Supplementary Table S1. Full list of fold-changes of metabolites identified in AroG_209-9_-expressing tomato line by GC-MS analysis.

Supplementary Table S2. Full list of fold-changes of metabolites identified in AroG_209-9_-expressing tomato line by UPLC/qTOF-MS analysis.

Supplementary Table S3. List of putative metabolites identified in AroG_209-9_-expressing tomato line by UPLC/qTOF-MS and MS-MS analysis.

Supplementary Table S4. Full list of fold-changes of metabolites identified in AroG_209-9_ expression tomato line by HPLC-PDA analysis.

Supplementary Fig. S1. Expression of the bacterial *AroG* gene in transgenic tomato.

Supplementary Fig. S2. Venn diagram compares the mass signal changes between three developing stages of peel and flesh tomato tissue detected by high-resolution LC-MS.

Supplementary Fig. S3. Correlation coefficient values of significant altered metabolites detected by GC-MS and LC-MS and unknown mass signals.

Supplementary Fig. S4. Correlation coefficient values of identify compounds detected by GC-MS and LC-MS.

Supplementary Data

## References

[CIT0001] Adato A Mandel T Mintz-Oron S 2009 Fruit-surface flavonoid accumulation in tomato is controlled by a SlMYB12-regulated transcriptional network. PLoS Genetics 5, 10.1371/journal.pgen. 1000777. 10.1371/journal.pgen.1000777 PMC2788616 20019811

[CIT0002] Baldwin E Scott J Shewmaker C Schuch W 2000 Flavor trivia and tomato aroma: biochemistry and possible mechanisms for control of important aroma components. Hortscience 35, 1013–1022

[CIT0003] Baldwin EA Scott JW Einstein MA Malundo TMM Carr BT Shewfelt RL Tandon KS 1998 Relationship between sensory and instrumental analysis for tomato flavor. Journal of the American Society for Horticultural Science 123, 906–915

[CIT0004] Ballester AR Molthoff J de Vos R 2010 Biochemical and molecular analysis of pink tomatoes: deregulated expression of the gene encoding transcription factor SlMYB12 leads to pink tomato fruit color. Plant Physiology 152, 71–84 19906891 10.1104/pp.109.147322 PMC2799347

[CIT0005] Bovy A de Vos R Kemper M 2002 High-flavonol tomatoes resulting from the heterologous expression of the maize transcription factor genes LC and C1. The Plant Cell 14, 2509–2526 12368501 10.1105/tpc.004218 PMC151232

[CIT0006] Buttery RG Ling LC 1993 Volatiles of tomato fruit and plant parts: relationship and biogenesis. Bioactive Volatile Compounds from Plants 525, 23–34

[CIT0007] Carrari F Baxter C Usadel B 2006 Integrated analysis of metabolite and transcript levels reveals the metabolic shifts that underlie tomato fruit development and highlight regulatory aspects of metabolic network behavior. Plant Physiology 142, 1380–1396 17071647 10.1104/pp.106.088534 PMC1676044

[CIT0008] Centeno DC Osorio S Nunes-Nesi A 2011 Malate plays a crucial role in starch metabolism, ripening, and soluble solid content of tomato fruit and affects postharvest softening. The Plant Cell 23, 162–184 21239646 10.1105/tpc.109.072231 PMC3051241

[CIT0009] Cooper DA 2004 Carotenoids in health and disease: Recent scientific evaluations, research recommendations and the consumer. Journal of Nutrition 134, 221–224 10.1093/jn/134.1.221S 14704323

[CIT0010] Dal Cin V Tieman DM Tohge T 2011 Identification of genes in the phenylalanine metabolic pathway by ectopic expression of a MYB transcription factor in tomato fruit. The Plant Cell 23, 2738–2753 21750236 10.1105/tpc.111.086975 PMC3226207

[CIT0011] Davidovich-Rikanati R Sitrit Y Tadmor Y 2007 Enrichment of tomato flavor by diversion of the early plastidial terpenoid pathway. Nature Biotechnology 25, 899–901 10.1038/nbt1312 17592476

[CIT0012] Deikman J Kline R Fischer RL 1992 Organization of ripening and ethylene regulatory regions in a fruit-specific promoter from tomato (*Lycopersicon esculentum*). Plant Physiology 100, 2013–2017 16653232 10.1104/pp.100.4.2013 PMC1075899

[CIT0013] Deikman J Xu R Kneissl ML Ciardi JA Kim KN Pelah D 1998 Separation of *cis* elements responsive to ethylene, fruit development, and ripening in the 5′-flanking region of the ripening-related E8 gene. Plant Molecular Biology 37, 1001–1011 9700072 10.1023/a:1006091928367

[CIT0014] Dexter R Qualley A Kish CM Ma CJ Koeduka T Nagegowda DA Dudareva N Pichersky E Clark D 2007 Characterization of a petunia acetyltransferase involved in the biosynthesis of the floral volatile isoeugenol. The Plant Journal 49, 265–275 17241449 10.1111/j.1365-313X.2006.02954.x

[CIT0015] Ding L Hofius D Hajirezaei MR Fernie AR Bornke F Sonnewald U 2007 Functional analysis of the essential bifunctional tobacco enzyme 3-dehydroquinate dehydratase/shikimate dehydrogenase in transgenic tobacco plants. Journal of Experimental Botany 58, 2053–2067 17463052 10.1093/jxb/erm059

[CIT0016] Doerfler H Lyon D Nägele T Sun X Fragner L Hadacek F Egelhofer V Weckwerth W 2013 Granger causality in integrated GC–MS and LC–MS metabolomics data reveals the interface of primary and secondary metabolism. Metabolomics 9, 564–574 23678342 10.1007/s11306-012-0470-0 PMC3651536

[CIT0017] Dudareva N Klempien A Muhlemann JK Kaplan I 2013 Biosynthesis, function and metabolic engineering of plant volatile organic compounds. New Phytologist 198, 16–32 23383981 10.1111/nph.12145

[CIT0018] Dudareva N Pichersky E 2008 Metabolic engineering of plant volatiles. Current Opinion in Biotechnology 19, 181–189 18394878 10.1016/j.copbio.2008.02.011

[CIT0019] Enfissi EM Barneche F Ahmed I 2010 Integrative transcript and metabolite analysis of nutritionally enhanced DE-ETIOLATED1 downregulated tomato fruit. The Plant Cell 22, 1190–1215 20435899 10.1105/tpc.110.073866 PMC2879742

[CIT0020] Farineau J Lavalmartin D 1977 Light versus dark carbon metabolism in cherry tomato fruits. 2. Relationship between malate metabolism and photosynthetic activity. Plant Physiology 60, 877–880 16660205 10.1104/pp.60.6.877 PMC542738

[CIT0021] Fernie AR Trethewey RN Krotzky AJ Willmitzer L 2004 Metabolite profiling: from diagnostics to systems biology. Nature Reviews. Molecular Cell Biology 5, 763–769 10.1038/nrm1451 15340383

[CIT0022] Fillati JA Sellmer J McCown B Haissig B Comai L 1987 *Agrobacterium*-mediated transformation and regeneration of *Populus* . Molecular and General Genetics 206, 192–199

[CIT0023] Fraser PD Enfissi EM Halket JM Truesdale MR Yu D Gerrish C Bramley PM 2007 Manipulation of phytoene levels in tomato fruit: effects on isoprenoids, plastids, and intermediary metabolism. The Plant Cell 19, 3194–3211 17933904 10.1105/tpc.106.049817 PMC2174704

[CIT0024] Fraser PD Pinto ME Holloway DE Bramley PM 2000 Technical advance: application of high-performance liquid chromatography with photodiode array detection to the metabolic profiling of plant isoprenoids. The Plant Journal 24, 551–558 11115136 10.1046/j.1365-313x.2000.00896.x

[CIT0025] Gillaspy G Ben-David H Gruissem W 1993 Fruits: a developmental perspective. The Plant Cell 5, 1439–1451 12271039 10.1105/tpc.5.10.1439 PMC160374

[CIT0026] Gleave AP 1992 A versatile binary vector system with a T-DNA organisational structure conducive to efficient integration of cloned DNA into the plant genome. Plant Molecular Biology 20, 1203–1207 1463857 10.1007/BF00028910

[CIT0027] Good X Kellogg JA Wagoner W Langhoff D Matsumura W Bestwick RK 1994 Reduced ethylene synthesis by transgenic tomatoes expressing S-adenosylmethionine hydrolase. Plant Molecular Biology 26, 781–790 7999994 10.1007/BF00028848

[CIT0028] Gutensohn M Klempien A Kaminaga Y 2011 Role of aromatic aldehyde synthase in wounding/herbivory response and flower scent production in different *Arabidopsis* ecotypes. The Plant Journal 66, 591–602 21284755 10.1111/j.1365-313X.2011.04515.x

[CIT0029] Halinska A Frenkel C 1991 Acetaldehyde stimulation of net gluconeogenic carbon movement from applied malic-acid in tomato fruit pericarp tissue. Plant Physiology 95, 954–960 16668078 10.1104/pp.95.3.954 PMC1077630

[CIT0030] Ibdah M Azulay Y Portnoy V 2006 Functional characterization of CmCCD1, a carotenoid cleavage dioxygenase from melon. Phytochemistry 67, 1579–1589 16563447 10.1016/j.phytochem.2006.02.009

[CIT0031] Kaminaga Y Schnepp J Peel G 2006 Plant phenylacetaldehyde synthase is a bifunctional homotetrameric enzyme that catalyzes phenylalanine decarboxylation and oxidation. Journal of Biological Chemistry 281, 23357–23366 16766535 10.1074/jbc.M602708200

[CIT0032] Key TJ Allen NE Spencer EA Travis RC 2002 The effect of diet on risk of cancer. Lancet 360, 861–868 12243933 10.1016/S0140-6736(02)09958-0

[CIT0033] Klee HJ 2010 Improving the flavor of fresh fruits: genomics, biochemistry, and biotechnology. New Phytologist 187, 44–56 20456053 10.1111/j.1469-8137.2010.03281.x

[CIT0034] Klee HJ Giovannoni JJ 2011 Genetics and control of tomato fruit ripening and quality attributes. Annual Review of Genetics 45, 41–59 10.1146/annurev-genet-110410-132507 22060040

[CIT0035] Lincoln JE Fischer RL 1988 Diverse mechanisms for the regulation of ethylene-inducible gene expression. Molecular and General Genetics 212, 71–75 3163768 10.1007/BF00322446

[CIT0036] Maeda H Shasany AK Schnepp J Orlova I Taguchi G Cooper BR Rhodes D Pichersky E Dudareva N 2010 RNAi suppression of arogenate dehydratase1 reveals that phenylalanine is synthesized predominantly via the arogenate pathway in *Petunia* petals. The Plant Cell 22, 832–849 20215586 10.1105/tpc.109.073247 PMC2861463

[CIT0037] Martens M Risvik E Martens H 1994 Matching sensory and instrumental analyses. In JR Piggott, A Paterson, eds, Understanding Natural Flavours Blackie, London

[CIT0038] Mattoo A Minocha S Minocha R Handa A 2010 Polyamines and cellular metabolism in plants: transgenic approaches reveal different responses to diamine putrescine versus higher polyamines spermidine and spermine. Amino Acids 38, 405–413 19956999 10.1007/s00726-009-0399-4

[CIT0039] Mintz-Oron S Mandel T Rogachev I 2008 Gene expression and metabolism in tomato fruit surface tissues. Plant Physiology 147, 823–851 18441227 10.1104/pp.108.116004 PMC2409049

[CIT0040] Mounet F Moing A Garcia V 2009 Gene and metabolite regulatory network analysis of early developing fruit tissues highlights new candidate genes for the control of tomato fruit composition and development. Plant Physiology 149, 1505–1528 19144766 10.1104/pp.108.133967 PMC2649409

[CIT0041] Ben Zvi MM Shklarman E Masci T Kalev H Debener T Shafir S Ovadis M Vainstein A 2012 PAP1 transcription factor enhances production of phenylpropanoid and terpenoid scent compounds in rose flowers. New Phytologist 195, 335–345 22548501 10.1111/j.1469-8137.2012.04161.x

[CIT0042] Negre-Zakharov F Long MC Doudareva N 2009 Floral scent and fruit aromas inspired by nature. New York: Springer Science+Business Media

[CIT0043] Osorio S Vallarino JG Szecowka M Ufaz S Tzin V Angelovici R Galili G Fernie AR 2013 Alteration of the interconversion of pyruvate and malate in the plastid or cytosol of ripening tomato fruit invoke diverse consequences on sugar, yet similar effects on cellular organic acid, metabolism and transitory starch accumulation. Plant Physiology 161, 628–643 23250627 10.1104/pp.112.211094 PMC3561009

[CIT0044] Rogachev I Aharoni A 2012 UPLC-MS-based metabolite analysis in tomato. Methods in Molecular Biology 860, 129–144 22351175 10.1007/978-1-61779-594-7_9

[CIT0045] Saeed AI Sharov V White J 2003 TM4: a free, open-source system for microarray data management and analysis. Biotechniques 34, 374–378 12613259 10.2144/03342mt01

[CIT0046] Shaul O Galili G 1993 Concerted regulation of lysine and threonine synthesis in tobacco plants expressing bacterial feedback-insensitive aspartate kinase and dihydrodipicolinate synthase. Plant Molecular Biology 23, 759–768 8251629 10.1007/BF00021531

[CIT0047] Simkin AJ Schwartz SH Auldridge M Taylor MG Klee HJ 2004 The tomato carotenoid cleavage dioxygenase 1 genes contribute to the formation of the flavor volatiles β-ionone, pseudoionone, and geranylacetone. The Plant Journal 40, 882–892 15584954 10.1111/j.1365-313X.2004.02263.x

[CIT0048] Smith CA Want EJ O’Maille G Abagyan R Siuzdak G 2006 XCMS: processing mass spectrometry data for metabolite profiling using nonlinear peak alignment, matching, and identification. Analytical Chemistry 78, 779–787 16448051 10.1021/ac051437y

[CIT0049] Spitzer-Rimon B Farhi M Albo B 2012 The R2R3-MYB-like regulatory factor EOBI, acting downstream of EOBII, regulates scent production by activating ODO1 and structural scent-related genes in *Petunia* . The Plant Cell 24, 5089–5105 23275577 10.1105/tpc.112.105247 PMC3556977

[CIT0050] Spitzer-Rimon B Marhevka E Barkai O 2010 EOBII, a gene encoding a flower-specific regulator of phenylpropanoid volatiles’ biosynthesis in *Petunia* . The Plant Cell 22, 1961–1976 20543029 10.1105/tpc.109.067280 PMC2910970

[CIT0051] Stepansky A Galili G 2003 Synthesis of the *Arabidopsis* bifunctional lysine-ketoglutarate reductase/saccharopine dehydrogenase enzyme of lysine catabolism is concertedly regulated by metabolic and stress-associated signals. Plant Physiology 133, 1407–1415 14576281 10.1104/pp.103.026294 PMC281635

[CIT0052] Tieman D Taylor M Schauer N Fernie AR Hanson AD Klee HJ 2006 Tomato aromatic amino acid decarboxylases participate in synthesis of the flavor volatiles 2-phenylethanol and 2-phenylacetaldehyde. Proceedings of the National Academy of Sciences, USA 103, 8287–8292 10.1073/pnas.0602469103 PMC1472464 16698923

[CIT0053] Togari N Kobayashi A Aishima TR 1995 Relating sensory properties of tea aroma to gas chromatographic data by chemometric calibration methods. Food Research International 28, 485–493

[CIT0054] Tzin V Malitsky S Aharoni A Galili G 2009 Expression of a bacterial bi-functional chorismate mutase/prephenate dehydratase modulates primary and secondary metabolism associated with aromatic amino acids in *Arabidopsis* . The Plant Journal 60, 156–167 19508381 10.1111/j.1365-313X.2009.03945.x

[CIT0055] Tzin V Malitsky S Ben Zvi MM Bedair M Sumner L Aharoni A Galili G 2012 Expression of a bacterial feedback-insensitive 3-deoxy-d-arabino-heptulosonate 7-phosphate synthase of the shikimate pathway in *Arabidopsis* elucidates potential metabolic bottlenecks between primary and secondary metabolism. New Phytologist 194, 430–439 22296303 10.1111/j.1469-8137.2012.04052.x

[CIT0056] Verdonk JC Haring MA van Tunen AJ Schuurink RC 2005 ODORANT1 regulates fragrance biosynthesis in petunia flowers. The Plant Cell 17, 1612–1624 15805488 10.1105/tpc.104.028837 PMC1091778

[CIT0057] White PJ 2002 Recent advances in fruit development and ripening: an overview. Journal of Experimental Botany 53, 1995–2000 12324524 10.1093/jxb/erf105

[CIT0058] Whitney KD 2005 Linking frugivores to the dynamics of a fruit color polymorphism. American Journal of Botany 92, 859–867 21652467 10.3732/ajb.92.5.859

[CIT0059] Zanor MI Rambla JL Chaib J Steppa A Medina A Granell A Fernie AR Causse M 2009 Metabolic characterization of loci affecting sensory attributes in tomato allows an assessment of the influence of the levels of primary metabolites and volatile organic contents. Journal of Experimental Botany 60, 2139–2154 19346240 10.1093/jxb/erp086 PMC2682503

[CIT0060] Zhao L Lu L Zhang L Wang A Wang N Liang Z Lu X Tang K 2009 Molecular evolution of the E8 promoter in tomato and some of its relative wild species. Journal of Biosciences 34, 71–83 19430120 10.1007/s12038-009-0010-x

