# Supplementary material for: Tomato fruits expressing a bacterial feedback-insensitive 3-deoxy-d-arabino-heptulosonate 7-phosphate synthase of the shikimate pathway possess enhanced levels of multiple specialized metabolites and upgraded aroma
Source: J Exp Bot. 2013 Sep 4;64(14):4441–52. doi: 10.1093/jxb/ert250 (PMC3808321; doi:10.1093/jxb/ert250)
Supplement: Supplementary Data [file supp_64_14_4441__index.html]

Tomato fruits expressing a bacterial feedback-insensitive 3-deoxy-d-arabino-heptulosonate 7-phosphate synthase of the shikimate pathway possess enhanced levels of multiple specialized metabolites and upgraded aroma — Tomato fruits expressing a bacterial feedback-insensitive 3-deoxy-d-arabino-heptulosonate 7-phosphate synthase of the shikimate pathway possess enhanced levels of multiple specialized metabolites and upgraded aroma — Supplementary Data 

# Tomato fruits expressing a bacterial feedback-insensitive 3-deoxy-d-arabino-heptulosonate 7-phosphate synthase of the shikimate pathway possess enhanced levels of multiple specialized metabolites and upgraded aroma

## 

Data files

**Files in this Data Supplement:**

- Supplementary Data - Supplementary Data
- Supplementary Data - Supplementary Data
