# Supplementary material for: Tomato fruits expressing a bacterial feedback-insensitive 3-deoxy-d-arabino-heptulosonate 7-phosphate synthase of the shikimate pathway possess enhanced levels of multiple specialized metabolites and upgraded aroma
Source: J Exp Bot. 2013 Sep 4;64(14):4441–52. doi: 10.1093/jxb/ert250 (PMC3808321; doi:10.1093/jxb/ert250)
Supplement: Supplementary Data [file supp_ert250_jexbot100875_file002.pdf]

Fig. S1

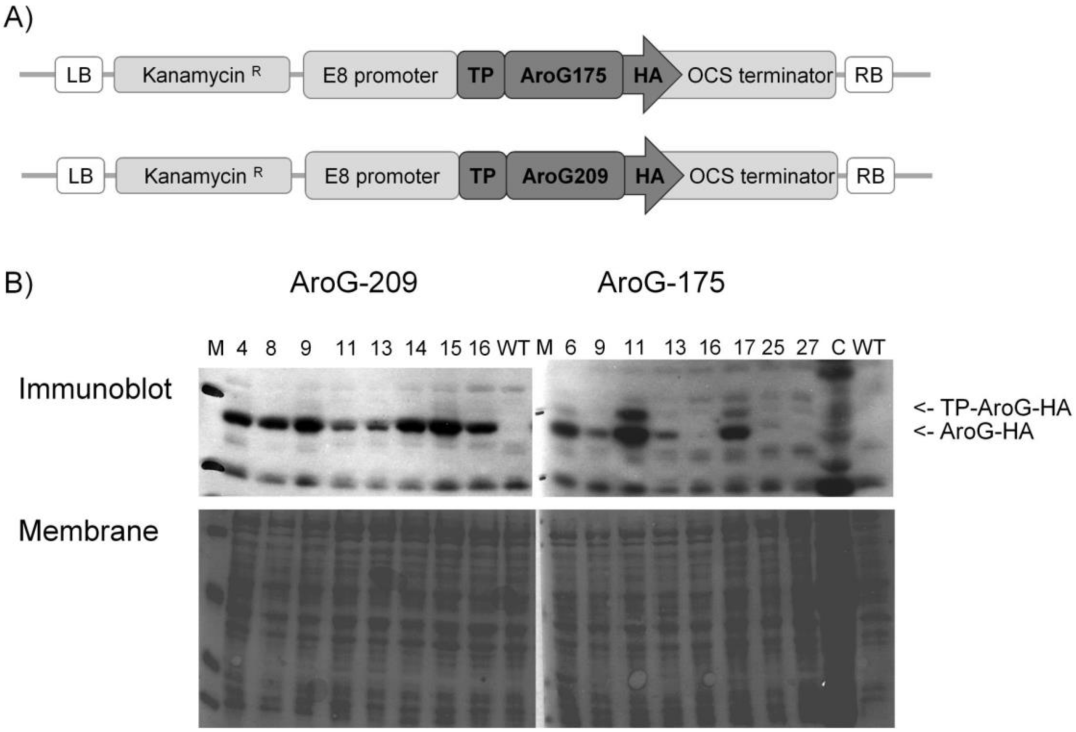

<u>Supplementary Fig. S1.</u> Expression of the bacterial *AroG* gene in transgenic tomato. A) Schematic diagram of the chimeric *AroG* gene. E8 fruit specific promoter; TP, plastid transit peptide; AroG, the bacterial *DAHPS* including three variance: mutant variances in amino acid located at 175 and 209 respectively. HA, three copies of the hemagglutinin epitope tag; OCS, octopine synthase terminator. B) Immunoblot analysis of protein extracts from independently transformed lines reacted with anti HA antibodies. The upper band (TP-AroG-HA) represents the precursor polypeptide (as detected in $AroG_{175\text{-}11}$), while the lower band (AroG-HA) represents the mature polypeptide. Loading WT showed comparable levels of stained proteins in the different lanes. Abbreviations: M, marker and C, WT.

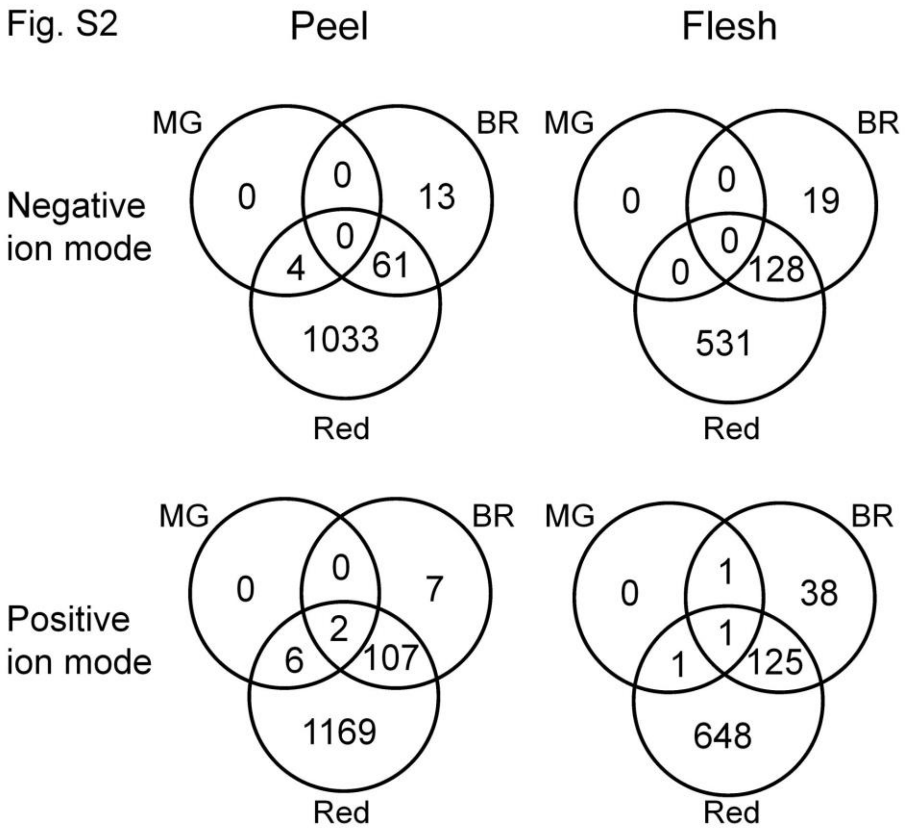

<u>Supplementary Fig. S2.</u> Venn diagram compares the mass signals changes between three developing stages of peel and flesh tomato tissue detected by high-resolution LC-MS. A Venn diagram is showing the relations between the differential mass signals in at least one out of three developing stages in two ionization modes in *AroG*$_{209\text{-}9}$ transgenic tomato peel and flesh tissues. The total number of mass signals that were significantly altered and used for the Venn diagrams are: (i) peel negative ion mode - 1,111; (ii) flesh negative ion mode - 678; (iii) peel positive ion mode - 1291 and (iv) flesh positive ion mode - 841. MG: mature green; BR: breaker; Red: ripe red.

Fig. S3

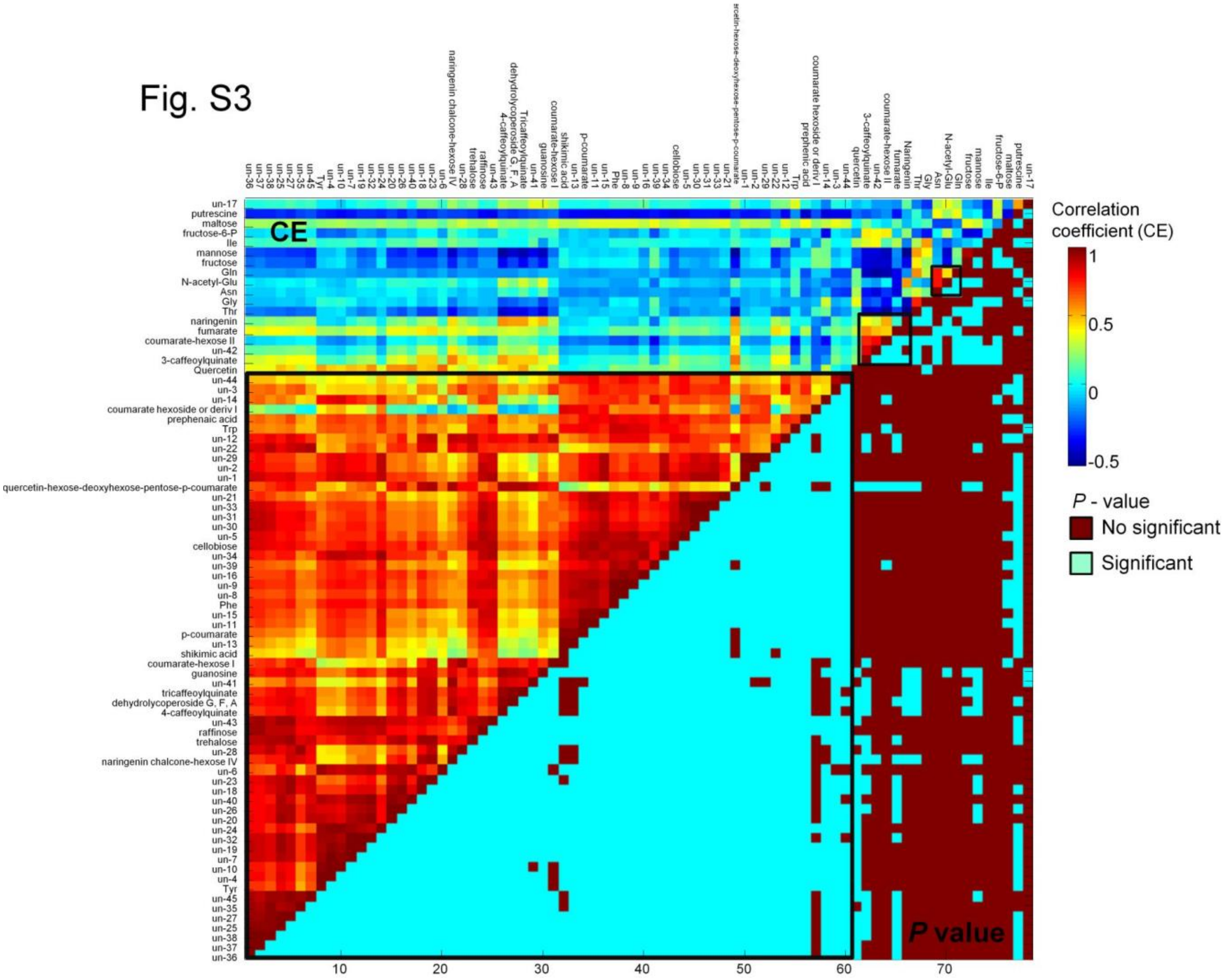

<u>Supplementary Fig. S3.</u> Correlation coefficient values of significant altered metabolites detected by of GC-MS (18) and LC-MS (15) and unknown mass signals (45). Metabolites and mass signals levels were identified and measured of three developing stages in two ion modes in *AroG*$_{209\text{-}9}$ transgenic tomato peel and flesh compare to WT (n=5-6). The upper triangle matrix presents Pearson's correlation (scale -0.5 to 1) and lower triangle matrix presents *P*-value of the correlation coefficient test (light blue present *P* value <0.01 and brown present non-significant). The heat map was performed using MATLAB software.

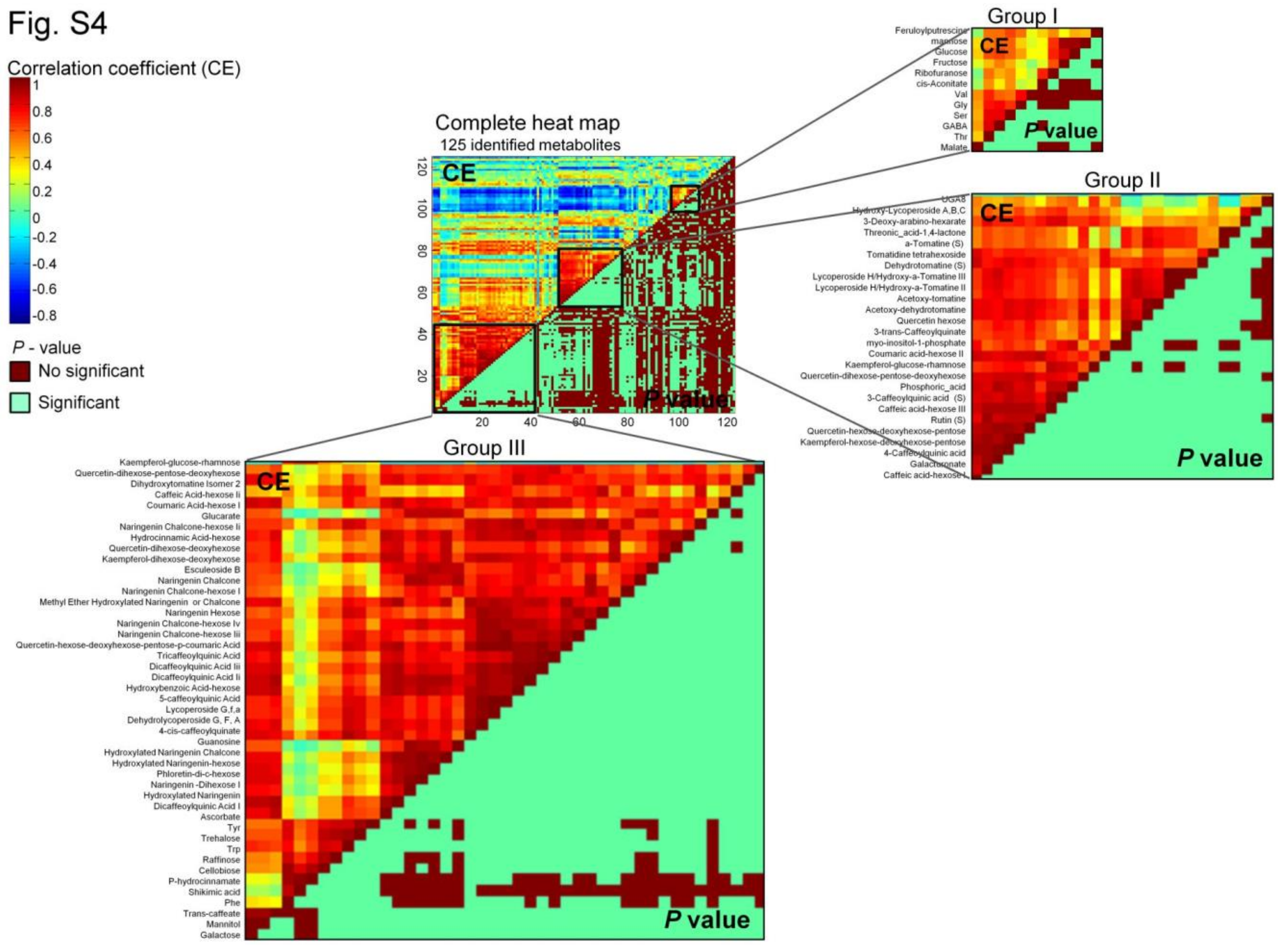

Supplementary Fig. S4. Correlation coefficient values of identify compounds detected by of GC-MS (61) and LC-MS (64). Metabolites and mass signals levels were identified and measured of three developing stages in two ion modes in *AroG*$_{209-9}$ transgenic tomato peel and flesh compare to WT (n=5-6);. The upper triangle matrix presents Pearson's correlation (scale -0.8 to 1) and lower triangle matrix presents *P*-value of the correlation coefficient test (light blue present *P* value <0.01 and brown present non-significant). The heat map was performed using MATLAB software.
